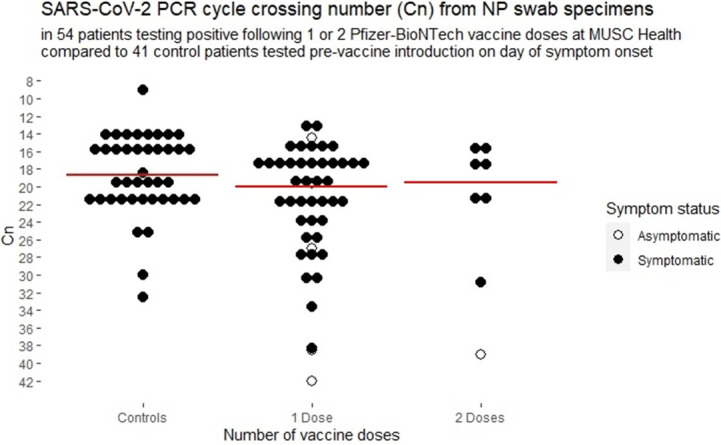# Laboratory-Confirmed SARS-CoV-2 Following One or More Doses of mRNA Vaccine Among Care Team Members at an Academic Health System

**DOI:** 10.1017/ash.2021.30

**Published:** 2021-07-29

**Authors:** Scott Curry, Cassandra Salgado, Krutika Kuppalli, Patricia Nickerson, Morgan Schrader, Danielle Scheurer, Julie Hirschhorn

## Abstract

**Background:** Medical University of South Carolina Health began vaccinating care team members December 15, 2020, with the Pfizer-BioNTech SARS-CoV-2 mRNA vaccine. We report surveillance data for cases diagnosed following vaccination. **Methods:** Care team members (CTMs) diagnosed with COVID-19 following SARS-CoV-2 vaccination were self-identified during online electronic contact-tracing surveys. Demographic data, symptoms, and dates of symptoms were recorded. CTMs testing positive at MUSC were linked to viral burden data from nasopharyngeal swabs tested on Abbott PCR platforms. **Results:** As of January 31, 2021, 111 CTMs tested positive for SARS-CoV-2 following vaccination: 99 and 12 after 1 and 2 doses, respectively, at medians of 10 days (range, 1–22) and 5 days (range, 1–16), respectively, from vaccination to testing. Of 2 cases that tested positive >14 days from dose 2, CTMs had symptom onset at 4 and 12 days from dose 2. Among CTMs reporting symptoms, 104 did so after a median of 7 days (mean 6.3, range −23 to +22) from vaccination to symptom onset, with 8 reporting symptoms before vaccination, 9 on the day of vaccination, and 3 CTMs at 1 day after vaccination, 6 CTMs at 2 days after vaccination, and 11 CTMs at 3 days after vaccination. Overall, 86 (78%) of 111 were female and 95 (86%) of 111 were white. The median age was 44 years (range, 22–71). Clinical job roles were most frequently nurses (n = 31, 28%), physicians or physician extenders (n = 19, 17%), and CTMs with no patient contact (n = 21, 19%). Assessment by the contact-tracing team assigned sources as household clusters (n = 22, 23%), local transmission (n = 21, 22%), occupational acquisition from coworkers (n = 11, 12%), travel related (n = 9, 10%), and unknown (n = 32, 34%). Only 32 (32%) CTMs were compliant with physical distancing. Among 104 CTMs reporting symptoms, cough (75%), headache (71%), rhinorrhea (63%), myalgia (60%), sore throat (48%), anosmia (44%), and subjective fever (40%) were the most commonly reported symptoms. Among 54 symptomatic CTMs with available viral-load data, the median and mean cycle numbers (Cn) were 19.98 and 21.91, respectively, for samples tested a median 3 days from symptom onset. Asymptomatic and symptomatic CTMs had a median Cn of 30.1 vs 20.9, respectively (p <0.001) and overall 50% of vaccinated CTMs had Cn >20, with no significant effect seen by vaccine dose (Figure 1). **Conclusions:** Most COVID-19 cases following vaccination occurred in CTMs with infection incubating prior to vaccination. No significant attenuation of viral load is apparent among vaccinated CTMs with COVID-19, but asymptomatic CTMs diagnosed with COVID-19 following vaccination appear to have resolved infections. Our data reinforce the need to adhere to public health measures by people who have been vaccinated.

**Funding:** No

**Disclosures:** None

**Figure 1.**